# Text Mining Analysis to Evaluate Stakeholders’ Perception Regarding Welfare of Equines, Small Ruminants, and Turkeys

**DOI:** 10.3390/ani9050225

**Published:** 2019-05-08

**Authors:** Emanuela Dalla Costa, Vito Tranquillo, Francesca Dai, Michela Minero, Monica Battini, Silvana Mattiello, Sara Barbieri, Valentina Ferrante, Lorenzo Ferrari, Adroaldo Zanella, Elisabetta Canali

**Affiliations:** 1 Dipartimento di Medicina Veterinaria, Università degli Studi di Milano, 20133 Milano, Italy; francesca.dai@unimi.it (F.D.); michela.minero@unimi.it (M.M.); monica.battini@unimi.it (M.B.); silvana.mattiello@unimi.it (S.M.); sara.barbieri@unimi.it (S.B.); 2 Istituto Zooprofilattico Sperimentale della Lombardia e dell’Emilia Romagna-Sezione diagnostica di Bergamo, 24125 Bergamo, Italy; vito.tranquillo@izsler.it; 3 Dipartimento di Scienze e Politiche Ambientali, Università degli Studi di Milano, 20133 Milano, Italy; valentina.ferrante@unimi.it (V.F.); lorenzo.ferrari@unimi.it (L.F.); 4 Department of Preventive Veterinary Medicine and Animal Health (VPS), University of São Paulo, São Paulo, SP 05508-270, Brazil; adroaldo.zanella@usp.br; 5 Dipartimento di Scienze Agrarie e Ambientali - Produzione, Università degli Studi di Milano, Territorio, Agroenergia, 20133 Milano, Italy; elisabetta.canali@unimi.it

**Keywords:** animal welfare, stakeholder perception, text mining, horse, donkey, goat, sheep, turkey

## Abstract

**Simple Summary:**

Consumers are currently more sensitive regarding the way animals are kept and handled and, in general, have increased their awareness towards animal welfare. There is a close link between welfare of animals and stakeholders’ perception of their needs. This study aimed at investigating stakeholders’ perception of the welfare of equines, small ruminants, and turkeys using a text mining approach to analyze their answers to open-ended questions. A total of 270 surveys were collected from respondents of 32 different countries. Independently from the species, respondents considered that an animal needs appropriate nutrition to be fit, healthy, and productive. The “environment” was considered particularly important for turkey stakeholders, whereas housing factors were relevant for goat stakeholders. Horse stakeholders also considered “exercise”, “pasture” and “proper training” important. Although the sample was too small to analyze variation in welfare perception among stakeholders of different species, text mining analysis seems to be a promising method to investigate stakeholders’ perception of animal welfare, as it emphasizes their real perception, without the constraints deriving by close-ended questions.

**Abstract:**

Welfare of animals significantly depends on how stakeholders perceive their needs and behave in a way to favor production systems that promote better welfare outcomes. This study aimed at investigating stakeholders’ perception of the welfare of equines, small ruminants, and turkeys using text mining analysis. A survey composed by open-ended questions referring to different aspects of animal welfare was carried out. Text mining analysis was performed. A total of 270 surveys were filled out (horses = 122, sheep = 81, goats = 36, turkeys = 18, donkeys = 13). The respondents (41% veterinarians) came from 32 different countries. To describe welfare requirements, the words “feeding” and “water” were the most frequently used in all the species, meaning that respondents considered the welfare principle “good feeding” as the most relevant. The word “environment” was considered particularly important for turkeys, as well as the word “dry”, never mentioned for other species. Horses stakeholders also considered “exercise” and “proper training” important. Goat stakeholders’ concerns are often expressed by the word “space”, probably because goats are often intensively managed in industrialized countries. Although the sample was too small to be representative, text mining analysis seems to be a promising method to investigate stakeholders’ perception of animal welfare, as it emphasizes their real perception, without the constraints deriving by close-ended questions.

## 1. Introduction

Citizen concern regarding animal welfare has been increasing in many parts of the world [1]; in general, consumers are more sensitive regarding the way farm animals are kept and handled [2,3,4]. Harper and colleagues reported that “animal welfare is used by consumers as an indicator of important product attributes, such as safety and the impact on health” [3]. However, the welfare of animals significantly depends on how stakeholders perceive their needs and behave with them. Indeed, stakeholders’ perception and animal welfare are closely linked because if stakeholders do not perceive that their animals have certain needs, it is less likely that they will protect them. Different stakeholders, such as farmers, veterinarians, and animal owners, may have a different perception regarding what an animal needs to be fit, healthy, and productive. Therefore, identifying different stakeholders’ perception is necessary to lead to improvement of animal welfare at farm level. 

Stakeholders’ engagement was fostered during the AWIN (Animal Welfare Indicators) research project, an EU-funded research project aimed to improve animal welfare through the development of practical on-farm animal welfare assessment protocols [5]. During the AWIN project (2011–2015), stakeholders’ input was proactively sought in several participatory activities with the aim to increase the acceptability of the project outcomes, but also to identify potential barriers to the practical application of the protocols [6,7]. As a first step of the AWIN project, stakeholders’ opinion on animal welfare was gained through an open-ended online survey [6,8,9]. 

Surveys have often been used to assess perceptions on welfare issues in different animal species, such as small ruminants [10,11], cattle [2,10] and horses [12,13,14,15,16,17,18,19]. The use of online surveys in animal welfare science presents several advantages: They are quick and easy to create, their creation and distribution is cheap (they can be shared via email, websites, and social networks), and they allow researchers to reach a wide number of respondents all around the world. 

The response rate and a possible bias in the sample of respondents (e.g., people without internet access cannot answer) are recognized issues when using online surveys to collect stakeholders’ opinion [20]. Other possible problems are: incomplete questionnaires resulting in incomplete data collection [21], and surveys with answers provided (closed-ended surveys) could not reflect all the possible answers of the respondents [1]. Open-ended questions may overcome this last problem, as they allow respondents to use their own words to express their feelings, attitudes and understanding of the subject and to include more information and details [1]. Therefore, surveys based on open-ended questions permit researchers to better understand the respondents’ true feelings on a topic and/or an issue. One of the possible difficulties of open-ended questionnaires is transforming the answers in statistical data, for further analysis. In order to solve this issue, new statistical techniques were developed, such as opinion mining [22,23] and text mining [24,25].

Text mining analysis is an approach to text analysis that has been frequently used in different scientific fields, from economics to history and from social science to biology [26,27,28,29,30]. The term text mining refers to a “process of distillation of useful information from a text” [26]. It is a set of quantitative methods that use the words present in a text as “units” of analysis. It applies to different types of texts (e.g., books, tweets, mails, open-ended surveys). The text is first “tokenized”, i.e., reduced to a sequence of simple terms deprived of those words (stop words) that serve the sensible and comprehensible definition of a period (e.g., article, adverb, number, punctuation). Stop words are commonly found in documents; they add little value to understanding the meaning of a sentence. For instance, in the sentence: “we have to ensure movement freedom, it’s compulsory” the words: “we, have, to, it’s” are considered stop words. This document called Corpus is then transformed into a term document matrix (TDM), a matrix that shows for each single term how many times it appears in a single document. From this matrix, all the types of textual analysis are obtained, including: Word frequency (how many times the same word appears in the same textual document), word association (it refers to the term pairings), text clustering (the application of cluster analysis to textual documents), sentiment analysis (systematically identify, extract, quantify, and study affective states and subjective information), and many more [25,26,31]. 

This work is a first attempt to approach the issue of stakeholders’ perception of animal welfare using text mining analysis. The study aimed at investigating, through open-ended questions, stakeholders’ perception of what equine, small ruminants, and turkeys need to be fit, healthy, and productive, and which signs would be observed using a text mining approach to analyze the answers.

## 2. Materials and Methods 

### 2.1. Web Survey

For each species (horses, donkeys, sheep, goats, and turkeys), a survey composed of 14 open-ended questions (max. 150 characters for each answer) referring to different aspects of animal welfare was carried out. The target population included in the present study refers in particular to farmers, veterinarians, and animal owners in order to collect information regarding animal welfare of different species (horses, donkeys, sheep, goats, and turkeys). To take the survey, respondents were required to be over the age of 18. The survey, translated in five languages (English, French, Italian, Portuguese, and Spanish), was published on a web platform (http://www.questionari.unimi.it/awin/), and it was freely accessible for 15 months (December 2012–March 2014). The survey link was anonymous, as this option was reported to be the easiest way to disseminate the survey and gather answers. The web link was shared via email, social networks, and hosted in websites of several academic and international organizations (such as FAO, International Society for Equitation Science, Italian Equestrian Federation, Istituto Zooprofilattico Sperimentale dell’Abruzzo e del Molise “Giuseppe Caporale”) in order to reach different stakeholders. 

Questions were related to four main topics: (1) needs (what do animals need to be fit, healthy, and productive?); (2) behavior (how might an animal behave/react in response to the following situations: noise, isolation, presence of known/unknown animal/person?) (3) emotions (how might an animal feel in response to the following situations: noise, isolation, presence of known/unknown animal/person?); and (4) welfare indicators (looking at your neighbor’s animals, which signs would you observe to assess: accommodation, feeding, health, manifestation of normal and abnormal behavior?). All the questions included in the survey are presented in Supplementary Table S1. In order to investigate stakeholders’ perception of welfare, in this manuscript, we focused only on welfare and its measures as reported by stakeholders; for this reason, only the answers referring to the six questions related to topics 1 and 4 (Table 1) are presented. For topic 4, due to the number of questionnaires collected, only horses were considered.

### 2.2. Text Mining Analysis

Data were imported into Microsoft Excel (Microsoft Corporation, Redmond, USA, 2010) and then a Text mining analysis was performed using R statistical software [32] by “tm” R package version 0.7-6 (https://CRAN.R-project.org/package=tm) [31]. A “document” was defined as every answer to single questions. A pre-process of answers was undertaken, to get the Corpus for each single question: English translation from other languages (Italian, Spanish, Portuguese, and French), exclusion of words according to a preselection list of so-called “stop-words”, cleaning (e.g., numbers, punctuation). For the question “In your opinion, what do animals need to be fit, healthy, and productive?” the term document matrix was performed, then a frequency words analysis was carried out, meaning that the most used words were identified. For the questions “Looking at your neighbour’s animal, which signs would you observe to assess accommodation, feeding, health, manifestation of normal and abnormal behaviour?”, only answers for horses were considered in the present paper as, for the other species, the number of respondents was too limited to allow meaningful analysis. The TDM initially was performed, then a frequency words analysis, followed by the term association analysis was carried out. Words with similar meaning (i.e., feed, food and feeding, etc.) were merged together. In the text mining analysis, terms association is like a correlation; it means that there is a co-presence of terms in the same document. The analysis of association therefore measures the co-presence between a term of interest defined by us and all the other words. Association study refers to the term pairings (when the term *x* appears, the other term *y* is associated with it), and it is not directly related to the frequency of terms. Unlike statistical correlation, text mining association ranges between 0 and 1: Score 1 means that two words always appear together in the documents, while a score approaching 0 means the terms seldom appear in the same document. The more often two terms appear in the same document, the stronger their association is. For each question, the association study was performed between the most frequent word and all the other terms. Associations with a value greater than or equal to 0.20 were considered relevant. 

## 3. Results

A total of 270 surveys were properly filled out (122 for horses, 81 for sheep, 36 for goats, 18 for turkeys, and 13 for donkeys). The respondents came from 32 different countries spread in the five continents (Table 2), but the majority of them were from European countries (70%), among whom 41% were Italian. 

In general, stakeholders were mainly veterinarians (41%), but some differences in stakeholders’ role were observed among species. For example, for horses, most of the respondents were private owners and trainers (34% and 13%, respectively), whereas for goats, they were mainly farmers (47%); for turkeys, contract farmers (11%) were also included (Table 3). 

Gender of respondents was balanced in all species (Table 4), except for horses (85% women). Most of the respondents (77%) were aged between 31 and 60 years, while 17% and 6% of respondents were under 30 and over 60, respectively. 

### 3.1. In Your Opinion, What do Animals Need to be Fit, Healthy, and Productive?

**Figure 1:**
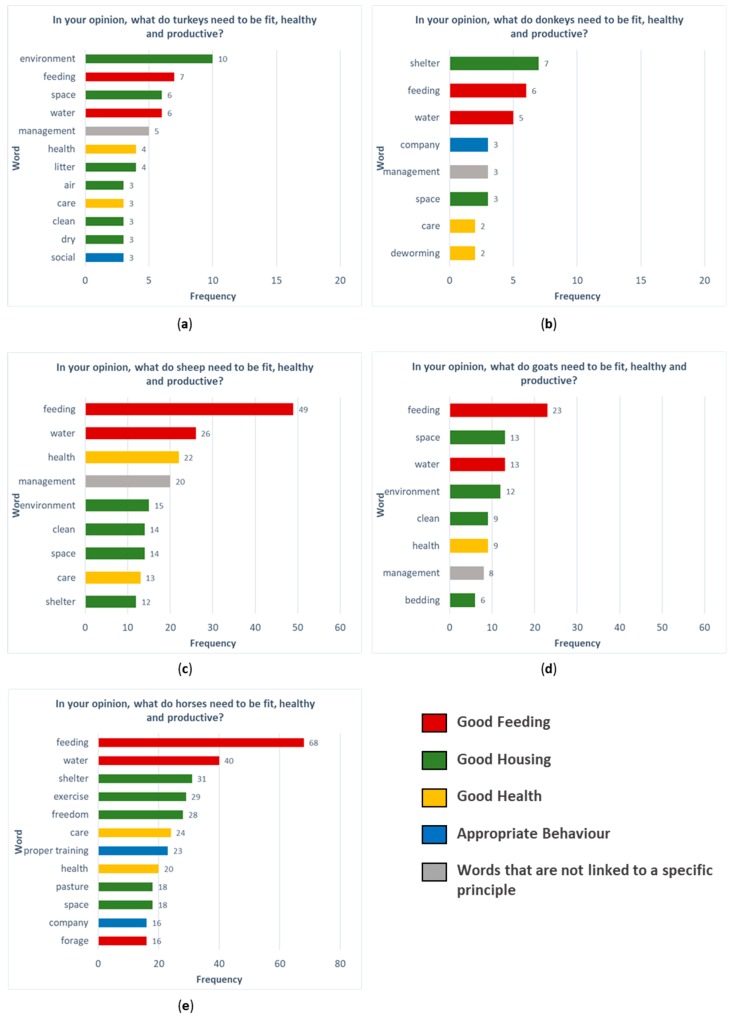
The graphs report the frequency of words used to answer the question “In your opinion, what do turkeys (**a**), donkeys (**b**), sheep (**c**), goats (**d**), and horses (**e**) need to be fit, healthy, and productive?” To highlight the association between each word and welfare principles [34], different colors are used: Red for “good feeding”, green for “good housing”, yellow for “good health”, blue for “appropriate behaviour”; grey is used for the words that are not linked to a specific principle.

The results of the word frequency analysis on the answers of the stakeholders of each species to the question “In your opinion, what do animals need to be fit, healthy and productive?” are reported in Figure 1. Words are presented according to the four welfare principles identified by Welfare Quality^®^ [33].

To describe welfare requirements, the words “feeding” and “water” were the most frequently used in all the species, meaning that respondents considered the welfare principle “good feeding” as the most relevant to guarantee fitness, health, and production. Horse stakeholders used the word “forage” to highlight the need of fiber in the horse diet. As for the principle “good housing”, “clean”, “shelter”, “environment”, “space”, and “bedding/litter” appeared to be of primary importance. The word “environment” was considered particularly important for turkeys, as well as the word “dry”, which is never mentioned by stakeholders of other species. Horse stakeholders also considered “exercise” and the presence of “pasture” important. The principle “good health” was represented by words like “care” and “health”. Interestingly, for donkeys, the word “deworming” was used. The welfare principle “appropriate behaviour” was addressed by equine and turkey stakeholders using the words “company/social”; for horses, “proper training” was also mentioned. Interestingly, small ruminants’ stakeholders did not use any word referring to this welfare principle. Stakeholders of different species, except horses, frequently used the word “management”, which is a general term, not clearly linked with a specific welfare principle.

### 3.2. Looking at Your Neighbor’s Horse, Which Signs would You Observe to Assess Accommodation, Feeding, Health, Manifestation of Normal and Abnormal Behaviour?

**Figure 2:**
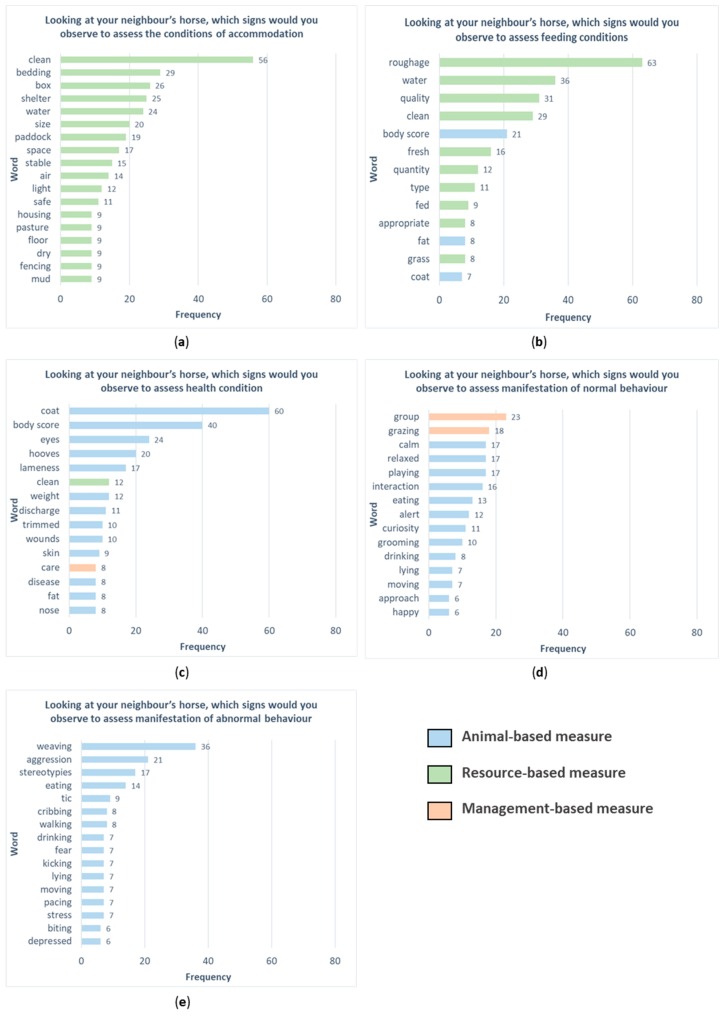
The graphs report the frequency of words for each of the five questions: “Looking at your neighbour’s horse, which signs would you observe to assess the conditions of accommodation (**a**), feeding conditions (**b**), health condition (**c**) manifestation of normal behaviour, (**d**) and manifestation of abnormal behaviour (**e**)?”. Different colors highlight the type of measure (animal-, resource- or management-based).

The results of the word frequency analysis of these five questions for horses are reported in Figure 2. An example of answer to the question “looking at your neighbor’s horse, which signs would you observe to assess the condition of accommodation?” was: “Presence and cleanliness of bedding, repair and maintenance of buildings, pastures and equipment”. The most frequent word used by horse stakeholders to evaluate the conditions of accommodation (Figure 2a) was “clean”, followed by “bedding”, “box”, “shelter”, and “water”. The text mining association showed that the term “clean” was associated with words referring to resource-based measures such as “floors” (0.33), “manger” (0.33), “drinkers” (0.32), “walls” (0.32), and “water” (0.31). 

An example of an answer to the question “Looking at your neighbour’s horse, which signs would you observe to assess the feeding conditions?” was: “A lot of roughage. Free access to clean hay that is not dusty or mouldy. Or access to grazing”. The words “roughage”, “water”, “quality”, and “clean” were the most used by horse stakeholders to evaluate the feeding conditions (Figure 2b). The term “roughage” was associated with the words: “access” (0.52), “available” (0.32), and “daily” (0.32). Additionally, for this question, most of the words used by stakeholders referred to resource-based measures; three words (body score, fat, and coat) referred to animal-based measures.

“Body score and coat shine/condition. Horses posture, movement (lameness)” is an example of answer to the question “Looking at your neighbour’s horse, which signs would you observe to assess health conditions?“ The assessment of health condition (Figure 2c) was described by horse stakeholders with words such as “coat”, “body score”, “eyes”, and “hooves”. The term “coat” was associated with the words: “vet” (0.29), “clean” (0.26), and “worming” (0.26). 

An example of an answer to the question “Looking at your neighbour’s horse, which signs would you observe to assess the manifestation of normal behaviour?” was: “Horses are relaxed but still interested in their surroundings, they are eating hay or grazing, socializing together and allo-grooming too”. The most frequent word used (Figure 2d) was “group”, which can be considered as a management-based measure (even though it is strictly related to social behavior, which is an animal-based measure), followed by “grazing”, “calm”, “relaxed”, and “playing”. Most of the words used by the stakeholders referred to animal-based measures. The term “group” was associated with the words: “happy” (0.38) and “curious” (0.30). 

Finally, an example of answer to the question “Looking at your neighbour’s horse, which signs would you observe to assess the manifestation of abnormal behaviour?” was: “Biting on doors, walls, posts. Kicking at walls. Constant or frequent vocalizing. Weaving, windsucking, pacing up and down, holding head high, eyes wide open, withdrawn, uninterested in surroundings, not interested in food, laying ears flat back when people approach, sudden or explosive movements”. Abnormal behavior (Figure 2e) was described using words referring to animal-based measures such as “weaving”, “aggression”, and “stereotypies”. The term “weaving” was associated with the word “tic” (0.65), “eat” (0.31), and biting (0.27). 

## 4. Discussion

The aim of the present study was to investigate stakeholders’ perception of welfare of equids, small ruminants, and turkeys. Most surveys were filled out by horse stakeholders that seemed to present a higher interest in welfare compared to other species. Considering the geographic area and role of respondents, it could be hypothesized that survey sampling could have been affected by the country of origin and scientific background of the AWIN partners. Answers collected through an open-ended survey were analyzed using a text mining approach. To the authors’ knowledge, this is the first time that text mining analysis has been applied to investigate which words were the most used by stakeholders of different species to describe animal welfare. The results suggest that respondents’ perception regarding what an animal needs to be fit, healthy, and productive is primarily linked with the principle “good feeding” [33]. In fact, words like “feeding” and “water” were the most used by respondents. It is well known that the importance of appropriate nutrition is paramount to safeguarding welfare of farm animals [35], as it is a basic need to be healthy and productive. Feeding was also considered one of the most important attributes indicated in an Australian survey for sheep and goats by farmers and veterinarians [36], who represent the majority of our respondents for these two species. Welfare indicators related to nutrition were also considered among the most important welfare indicators in a survey on stakeholders’ perception of the welfare of extensively managed sheep [37]; in the same study, water provision was also included among the most important factors affecting welfare. For small ruminants, water was one of the most frequently used words for both species: For sheep, this is probably due to the fact that they are often managed in extensive systems, also in Mediterranean countries, where scarcity of water may actually be a limiting factor for animal welfare. For this reason, new indicators were developed and included in the AWIN welfare assessment protocol for goats [38] and in the AWIN welfare assessment protocol for sheep [39]. Unfortunately, although stakeholder differences in gender and role, as well as many other factors, may significantly affect the perception of animal welfare [33], the limited sample size of this survey did not allow an investigation in this sense, and the following results are pooled for all the questionnaires.

For horses, together with “feeding” and “water”, stakeholders recognized that providing “forage” is also necessary. The recognition of the importance of foraging is shown also by the use of other words such as “pasture”, “grazing”, “roughage”, and “grass”. Horses naturally spend the majority of their time grazing [40,41], and it is reported that foraging food with high fiber content is not only a physical need (80% of the time, the gastric tract should be filled), but also a psychological need for them [42]. In term of signs to evaluate feeding conditions, horse stakeholders referred mostly to resource-based indicators, emphasizing how the quality of feeding (“quality”, “clean”, “fresh”, and “appropriate”) is important. Nutrition is a critical component of horse health [43], and it is reported that good quality of feeding could be a reasonable target for decreasing the prevalence of pulmonary diseases in horses [44]. 

Words referring to the principle “good housing”, such as “shelter”, “space”, “clean”, “air”, and “bedding”, were frequently used by stakeholders of different species. This principle seems to be paramount for turkeys’ stakeholders: “environment” was the word most used. The fact that the environment is a key factor in maintaining poultry welfare is well known: Jones and colleagues demonstrated that a good ventilation, to control temperature and humidity, is even more important than density in determining poultry welfare [45]. Turkeys may show large behavioral adjustments as a response to inadequate environmental conditions, with consequences including gait deterioration, higher frequency of injuries, increased aggression levels or feather pecking occurrences [46].

Factors referring to “good housing” seemed more important for goats than for sheep stakeholders. In fact, words referring to this principle in goats are ranked higher than in sheep (Figure 1c,d), and they are cited for a total of 40 times in 36 questionnaires, whereas in sheep, they are mentioned only 55 times in 81 questionnaires. This is probably because in most industrialized countries, goats are often intensively managed, and they are kept indoors with only occasional access to pasture [47], and therefore, stakeholders’ concerns are often expressed by the word “space”. To evaluate the condition of the accommodation, horse stakeholders referred only to resource-based measures. In particular, they referred to “clean floors and walls”, “clean manger”, and “clean drinkers and water”. A lack of animal-based measures referring to the principle “good housing” of equines was already recognized [48]; for this reason, most of the indicators included in the AWIN welfare assessment protocol for horses [49] and in the AWIN welfare assessment protocol for donkeys [50] were resource-based measures such as “bedding” and “box dimensions”. 

For the principle “good health”, stakeholders used the same words (“health” and “care”) across species. For sheep, and even less for goats, these terms were not of primary importance. This is in contrast with the results of a survey carried out in Australia [36], where a health issue, parasite control, reached the highest importance value among stakeholders. However, it has to be noticed that the issue “parasite control” was suggested to the stakeholders from a fixed list, whereas in our case, there was no fixed list, and stakeholders were totally free to generate their own terms and to express their personal concerns. Donkey stakeholders considered also “deworming” as an important issue to address. In fact, it is well known that gastrointestinal parasites are common in equines, and they affect both their health and welfare [51,52,53]. Hence, the word “deworming” was used in association with the term “coat” by horse stakeholders to describe health conditions; this reflects that the presence of gastrointestinal parasites could affect the overall health of the subject.

Interestingly, no word was generated by small ruminant stakeholders referring to appropriate behavior. This principle was mentioned only by stakeholders of turkeys, donkeys, and horses. Particular emphasis was given to social behavior, with the words “company” and “social” used by equine and turkey stakeholders, respectively. Offering the possibility to horses to interact with conspecifics (e.g., “group” and “interaction”) was highlighted by respondents when asked to evaluate normal horse behavior. Despite the fact that stakeholders recognized that horses are highly social species, and thus, contact with conspecifics plays an important role in their welfare [54,55,56,57], single boxes, which restrict horses’ possibility of socialization, are still a standard housing system [58]. Another important aspect for horse stakeholders was “proper training”. Horses are kept for very different purposes, ranging from sport and leisure to use in animal-assisted therapies; for these reasons, they are exposed to training. Choosing appropriate training methods together with proper equipment is fundamental to guaranteeing not only good performances but also welfare of sport horses. In fact, several studies suggest that the use of inappropriate equipment such as bit, reins or nosebands may cause pain and stress [59,60,61] affecting horse response to training, reducing performances and causing the manifestation of potentially dangerous behaviors, and impairing horse welfare. 

The major constraint of this work, in common with other online questionnaires [20], is the limited sample: Although the survey was widely publicized, the number of respondents was too small to allow meaningful analysis of differences in welfare perceptions among different species’ stakeholders and among individual stakeholders’ characteristics within each species. In particular, for some species, such as turkeys and donkeys, few answers were collected. One possible explanation could be related to the advertising used to spread out the questionnaire. For example, of the 41 million donkeys present in the world, it is estimated that 96% are owned in developing countries [62], where internet connection is not so widely spread as in Europe. As for turkeys, another possible explanation is that the poultry supply chain consists of large numbers of animals concentrated in very few stakeholders. Furthermore, turkey stakeholders do not seem so willing to share their ideas regarding poultry welfare: This can be the reason most of the answers related to turkeys came from Italy, where AWIN researchers have been working to build a good relationship with the stakeholders and to focus their attention toward animal welfare. Finally, the lack of interest of the poultry sector in issues related to animal welfare could be linked to the fact that turkeys are seen much more in terms of productivity with respect to other species. However, increasing numbers are demanding animal welfare assurances for the products the poultry industry produces [63]. The industry must address these concerns or risk alienating clients and customers.

## 5. Conclusions

Stakeholders’ involvement is fundamental for any action intended to improve animal welfare; this work portrays the stakeholders’ perception, highlighting the need of proper dissemination of scientific knowledge. The words used by stakeholders were related to indicators included in the AWIN welfare assessment protocols. In stakeholders’ opinion, small ruminants need good feeding, but also a good environment, such as clean bedding, shelter, and enough space to be fit, healthy, and productive. For turkey stakeholders, the environment (dry and clean litter, clean air, space) is paramount to guaranteeing good welfare. Finally, stakeholders consider a horse fit, healthy, and productive if feeding is appropriate (including the possibility to eat roughage), at the same time allowing the possibility to interact with conspecifics and spend time at pasture. A limitation of this study was that the sample was too small to be representative of different stakeholders’ perception worldwide. However, text mining analysis seems to be a promising method to investigate stakeholders’ perception of animal welfare, as it emphasizes their real perception, without the constraints derived by close-ended questions. With a larger sample, it would be possible to undertake a more detailed analysis of welfare perception variations among species and to explore underlying reasons for these differences. 

## Figures and Tables

**Table 1:** List of the questions selected from the questionnaire whose results are reported in the manuscript.

Question	Type of Question
In your opinion, what do sheep/goats/turkeys/donkeys/horses need to be fit, healthy and productive?	Open text (max 150 characters)
Looking at your neighbour’s horse, which signs would you observe to assess ^1^	
- The conditions of accommodation ^1^	Open text (max 150 characters)
- Feeding conditions ^1^	Open text (max 150 characters)
- Health conditions ^1^	Open text (max 150 characters)
- The manifestation of normal behaviour ^1^	Open text (max 150 characters)
- The manifestation of abnormal behaviour ^1^	Open text (max 150 characters)

^1^ Only results for horses are reported for this topic.

**Table 2:** Percentage (%) of respondents for each geographic area.

Area	Respondents (N = 270)
Europe	70%
North America	12%
South and Central America	4%
Oceania	9%
Asia	3%
Africa	1%

**Table 3:** Stakeholder roles (%) for each species included in the survey (N = 270).

	Sheep (N = 81)	Goats (N = 36)	Horses (N = 122)	Donkeys (N = 13)	Turkeys (N = 18)	Overall (N = 270)
Veterinarian	56%	33%	35%	23%	50%	41%
Farmer	23%	47%	7%	62%	28%	21%
Contract farmer	-	-	-	-	11%	1%
Technician	21%	20%	11%	15%	11%	16%
Owner	-	-	34%	-	-	15%
Trainer	-	-	13%	-	-	6%

**Table 4:** Gender of respondents (%) for each species included in the survey (N = 270).

	Sheep (N = 81)	Goats (N = 36)	Horses (N = 122)	Donkeys (N = 13)	Turkeys (N = 18)	Overall (N = 270)
Male	60%	58%	15%	31%	56%	38%
Female	40%	42%	85%	69%	44%	62%

## Supplementary Materials

The following are available online at https://www.mdpi.com/2076-2615/9/5/225/s1, Table S1: List of the questions reported in the questionnaire.
