# Supplementary material for: Text Mining Analysis to Evaluate Stakeholders’ Perception Regarding Welfare of Equines, Small Ruminants, and Turkeys
Source: Animals (Basel). 2019 May 8;9(5):225. doi: 10.3390/ani9050225 (PMC6562437; doi:10.3390/ani9050225)
Supplement: Supplementary file 1 [file animals-09-00225-s001.pdf]

Table S1. List of the questions reported in the questionnaire.

**Questionnaire**
In your opinion, what do sheep/goats/turkeys/donkeys/horses need to be fit, healthy and productive?
Behavior: How does a sheep/goat/turkey/donkey/horse act and react in each of the following situations?
- In the presence of a noise, a sheep/goat/turkey/donkey/horse
- Isolated from the group, a sheep/goat/turkey/donkey/horse
- In the presence of animals / persons known, a sheep/goat/turkey/donkey/horse
- In the presence of unknown animals / persons, a sheep/goat/turkey/donkey/horse
Perception: How could a sheep/goat/turkey/donkey/horse feel in each of the following situations?
- In the presence of a noise, sheep/goat/turkey/donkey/horse
- Isolated from the flock, a sheep/goat/turkey/donkey/horse
- In the presence of animals / persons known, a sheep/goat/turkey/donkey/horse
- In the presence of unknown animals / persons, a sheep/goat/turkey/donkey/horse
Looking at your neighbour’s sheep/goat/turkey/donkey/horse, which signs would you observe to assess:
- The conditions of accommodation
- Feeding conditions
- Health conditions
- The manifestation of normal behavior
- The manifestation of abnormal behavior
